# Orofacial antinociceptive effect of a novel 2-amino-thiophene derivative and its possible targets of action

**DOI:** 10.1590/1807-3107bor-2025.vol39.099

**Published:** 2025-10-20

**Authors:** Alleson Jamesson da SILVA, Pablo Rayff da SILVA, Hugo Fernandes Oliveira PIRES, Arthur Lins DIAS, Cícero Francisco Bezerra FELIPE, Francisco Jaime Bezerra MENDONÇA-JUNIOR, Anuraj NAYARISSERI, Alan Ferreira ALVES, Marcus Tullius SCOTTI, Adriana Maria Fernandes de Oliveira GOLZIO, Ricardo Dias de CASTRO

**Affiliations:** (a) Universidade Federal da Paraíba – UFPB, School of Dentistry, Department of Clinical and Social Dentistry, João Pessoa, PB, Brazil.; (b) Universidade Federal da Paraíba – UFPB, Institute for Drugs and Medicines Research, João Pessoa, PB, Brazil.; (c) Universidade Estadual da Paraíba - UEPB, Laboratory of Molecular Synthesis and Vectorization, João Pessoa, PB, Brazil.; (d) Laboratory of in silico Research, Eminent Biosciences, Vijaynagar, Indore, India.; (e) Universidade Federal da Paraíba – UFPB, Laboratory of Cheminformatics, Institute for Drugs and Medicines Research, João Pessoa, PB, Brazil.

**Keywords:** Facial Pain, Analgesics, Pain Management, Thiophenes, Models, Animal

## Abstract

Orofacial pain impairs quality of life, and current therapies, limited in efficacy and associated with adverse effects, drive the search for new treatments. Thiophene derivatives exhibit remarkable therapeutic properties, including antinociceptive and anti-inflammatory activities, with recent studies demonstrating superior activity compared to commercial drugs, highlighting their relevance in the design of novel agents. This study investigated the antinociceptive effect of the thiophene derivative 2-[(4-diethylamino-benzylidene)-amino]-5,6,7,8-tetrahydro-4H-cyclohepta[b]thiophene-3-carbonitrile (7CN03) and its possible mechanisms of action. In vivo tests were performed on male mice (n = 6 per group), and nociception was induced by formalin, capsaicin, and glutamate 1 h after treatment. Facial rubbing was used as a parameter to measure nociceptive behavior. 7CN03 exhibited significant action during the neurogenic phase of the formalin test at different doses (1 mg/kg, 0.1 mg/kg, and 0.01 mg/kg), reducing nociceptive behavior by up to 56%. During the inflammatory phase, the 1 mg/kg dose exerted an antinociceptive effect, reducing nociceptive behavior by 32% (p < 0.05). In the glutamate test, 7CN03 blocked nociception by up to 90% (p < 0.001), and in the capsaicin test, it reduced nociceptive behavior by up to 74%. Molecular docking studies predicted higher binding affinity of 7CN03 for µ-opioid (-97.00 Kcal/mol), TRPV1 (-87.79 Kcal/mol), and NMDA (-104.86 Kcal/mol) receptors when compared with cocrystallized ligands. The findings suggest that the evaluated thiophene derivative exhibits an orofacial antinociceptive effect, with a mechanism of action likely mediated by opioid, transient receptor potential vanilloid, and glutamatergic receptors.

## Introduction

Orofacial pain is commonly reported as a comorbidity associated with disorders of the oral cavity or facial region and is characterized by the presence of pain in soft and/or mineralized tissues. The etiology of orofacial pain can be odontogenic or non-odontogenic. Non-odontogenic conditions are particularly challenging to diagnose, affecting muscles, joints, and nerves. They may also be associated with headaches, cervical pain, and rheumatic diseases such as fibromyalgia and rheumatoid arthritis.^1^

The prevalence of orofacial pain is 30.2% in developing countries, representing a significant share of the population, whose quality of life may be impaired by the biological consequences of orofacial pain. Orofacial pain is associated with functional limitations, such as limited mouth opening and pain during mastication, as well as psychological effects, including anxiety arising from uncertainty about symptom resolution.^2^

Unlike the spinal system, the conduction of nociceptive impulses through the trigeminal nerves is mediated by structures unique to the facial region, representing a distinct anatomical level not present elsewhere in the body.^3^ The uniqueness of the trigeminal system lies in the integration of nociceptive inputs at multiple levels, specifically in the brainstem, caudal subnucleus, and other components of the trigeminal nuclear complex, which also process epicritic touch and facial proprioception.^4^

Current therapies for orofacial pain include the use of analgesics or nonsteroidal or steroidal anti-inflammatory drugs. There is no ideal medication, however, for treating orofacial pain, as existing therapies are often limited or discontinued due to adverse effects such as gastrointestinal, hepatic, and renal disturbances or hypersensitivity reactions.^5^ In the particular case of opioids, pharmacological tolerance and dependence, which contribute to high morbidity and mortality rates in developed countries, highlight the need for lower doses or even the replacement of this class of drugs with other agents.^6^

Commercial nonsteroidal anti-inflammatory drugs (NSAIDs) such as tinoridine and tiaprofenic acid (used especially for the treatment of arthritis pain) contain a thiophene ring.^7^ Recent studies have demonstrated the importance of a series of thiophene-based compounds in drug design and the discovery of new anti-inflammatory agents. The vast majority of their planned and synthesized derivatives exhibit superior anti-inflammatory activity compared to reference NSAIDs, as evidenced by in vitro, in silico, and in vivo assays.^7,8^

Therefore, the thiophene derivative 7CN03 stands out as a promising agent for the treatment of orofacial pain, given its analgesic activity in the spinal system, as demonstrated by our previous studies. In screening assays aimed at identifying compounds with potential antinociceptive activity, preliminary studies have shown that the 7CN03 molecule exhibits antinociceptive effects when administered at low oral doses in animals experiencing formalin-induced nociception in the paw (unpublished results). These findings support the hypothesis that this compound has antinociceptive effects involving the trigeminal system.

Accordingly, this study aimed to investigate the orofacial antinociceptive effect of the 2-amino-thiophene derivative 7CN03 in mice using animal models of nociception that involve opioid receptors, transient receptor potential (TRP) channels, and glutamatergic receptors, as well as to elucidate the possible mechanisms of action of 7CN03through molecular docking evaluation.

## Methods

### In vivo tests

#### Study location, ethical aspects, and animal model

This experimental, non-clinical, controlled, and double-blind study utilized healthy adult Swiss albino mice (Mus musculus) weighing between 30 g and 40 g. The mice were maintained under controlled temperature conditions (21°C ± 2°C) and a 12-hour light/dark cycle, with food and water provided ad libitum. All animals were obtained from the Animal Production Unit of the Institute for Drug and Pharmaceutical Research (IPeFarm) at the Federal University of Paraiba, João Pessoa, state of Paraiba, Brazil.

The study protocol was approved by the Animal Research Ethics Committee of the Federal University of Paraíba (process no. 7123201123/2023). A reduced number of animals was considered for the experiments, and measures to control suffering or stress at all phases of experimentation were implemented in accordance with the ethical guidelines for animal research.

Sample size was calculated based on statistical criteria, considering the following parameters: two-tailed t-test, effect size equal to 2.444, significance level of 5% (α = 5%), and a power of 90%. These parameters indicated the need for five animals per experimental group, with an additional 20% allowance for potential loss, resulting in a requirement of six animals per group.

Details on reagents and substances, including resynthesis and comparison of spectral data of 7CN03, according to Rodrigues et al.^9^ and Cruz et al.,^10^ are provided as supplementary material.

#### Research design and experimental protocols for induction of orofacial nociception

Three models were tested to evaluate the orofacial antinociceptive activity of 7CN03: a) formalin-induced nociception, b) glutamate-induced nociception, and c) capsaicin-induced nociception. The experiments for each model were conducted in three independent groups, each comprising 30 animals equally distributed into five subgroups .

Animals were pretreated with morphine at 20 mg/kg (orally) or MK-801 (glutamate nociception induction test) at 15 μg/kg (i.p), 7CN03 at 1 mg/kg (orally), 7CN03 at 0.1 mg/kg (orally), 7CN03 at 0.01 mg/kg (orally), and saline (0.9% sodium chloride) (orally) in 20 μL of solution, 1 h before the induction of orofacial nociception. 7CN03 test dosages were determined based on its effects on the spinal system.

Data management and blinding methods were performed to reduce possible biases in the data collection and analysis. Test sequencing was randomized, with random allocation of animals. The preparation, conduct, and behavioral analysis were triple-blind and were carried out based on the protocol developed by Tomaz-Morais et al.^11^

#### Models of orofacial nociception induced by formalin, capsaicin, and glutamate

**Figure 1:**
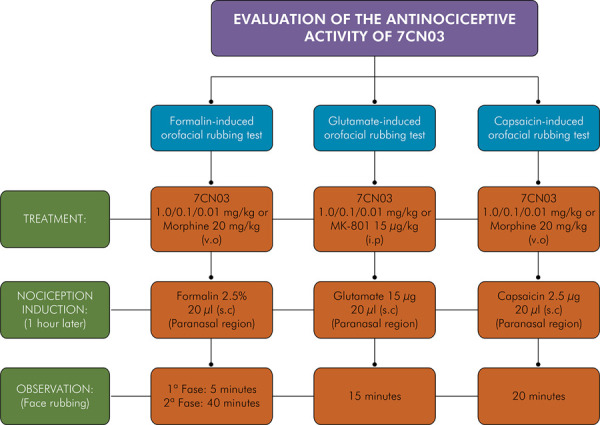
Flowchart of experimental protocols for induction of orofacial nociception.

Due to the unique sensory innervation of the face mediated by the trigeminal nerve, which transmits stimuli related to touch, pressure, temperature, and pain, the perinasal injection of nociceptive agents is well indicated in these models.

a.Formalin-induced orofacial rubbing test: According to the protocol described by Quintans-Júnior et al.,^12^ 20 μL of a 2.5% formalin solution (formaldehyde in distilled water) was administered subcutaneously into the lateral portion of the upper lip (paranasal region) of the mice 1 h after oral treatment with 7CN03, using a 27-gauge needle. Nociceptive behavior was observed for 40 min – 0 to 5 min in the neurogenic phase and 15 to 40 min in the inflammatory phase. Nociceptive activity was determined by recording the duration (in seconds) of rubbing toward the injected area on the ipsilateral front or rear paw.

b.Glutamate-induced orofacial rubbing test: The glutamate-induced nociception model was applied, as described by Beirith et al.^3^ In this model, 20 μL of a 15 μg glutamate solution was administered subcutaneously into the lateral portion of the right upper lip of mice with a 27-gauge needle 1 h after oral treatment with 7CN03. The animals were then observed for 15 min after glutamate administration. As in the previous test, nociceptive activity was determined by recording the friction time (in seconds) of the injected area with the ipsilateral front or hind paw.

c.Capsaicin-induced orofacial rubbing test: The capsaicin nociception induction model followed the protocol described by Quitans-Júnior et al.^12^ The tests were performed by administering 20 μL of a 2.5 μg capsaicin solution to the right upper lip of mice 1 h after oral treatment with 7CN03, followed by observation of nociceptive behavior (rubbing the injected area with the ipsilateral front or rear paw) for 20 min (Figure 1)

## In silico test

### Molecular docking

7CN03 was modeled using MarvinSketch v.23.14 software and optimized using the semi-empirical Merck Molecular Force Field (MMFF) method in Spartan v.14.^13,14^ The crystallographic structures of six targets involved in pain pathophysiology were selected from the Protein Data Bank (PDB) (Table 1), based on their roles in pain modulation. These targets include opioid receptors, which are primary mediators of analgesic responses, as well as transient receptor potential vanilloid 1 (TRPV1), transient receptor potential cation channel subfamily A (TRPA1), and N-methyl-D-aspartate (NMDA) receptors, which are critical for nociceptive signaling and central sensitization. As a comparison, agonists (opioid receptors) or antagonists (TRPV1, TRPA1, and NMDA) of each of the targets were selected.

**Table 1:** Targets and ligands used in molecular docking.

Target	PDB (ID)	Resolution	Ligands
µ-opioid receptor	8EF6 [3]	3.20 Å	Morphine
δ-opioid receptor	6PT3 [4]	3.10 Å	DPI-287
κ-opioid receptor	6B73 [5]	3.30 Å	MP1104
TRPV1	5IS0 [6]	3.43 Å	Capsazepine
TRPA1	7JUP [7]	3.05 Å	AM-0902
NMDA receptor	7EOQ [8]	3.50 Å	Esketamine

Values were expressed as mean and SD (n= 6, per group). Statistical analysis: One-way ANOVA followed by Tukey’s test, *p < 0.05, **p < 0.01, ****p < 0.001: (7CN) = vs. control and (morphine) = vs. control.

The Molegro Virtual Docker v.2013.6.0.1 software was used for molecular docking.^15^ All water molecules and cofactors were removed and the re-docking step was performed before molecular docking in order to evaluate the accuracy and reliability of the results through root mean square deviation (RMSD). RMSD is a necessary step to verify whether the algorithm was able to produce the correct docking pose, with RMSD values ≤ 2 Å regarded as satisfactory. RMSD values were not considered for the NMDA receptor (PDB: 7EOQ) because it did not have an available co-crystallized ligand, and esketamine was used as a positive control.

The simulation was conducted using default settings. The MolDock score function was used to evaluate the ligand poses, considering internal energy, hydrogen bonding, and torsional energy contributions. It is important to note that the MolDock score is not expressed in chemically relevant energy units (e.g., kcal·mol^-1^ or kJ·mol^-1^). Instead, these scores serve as relative indicators of binding affinity, where lower (more negative) values suggest stronger ligand-receptor interactions.^16,17^

Twenty runs were performed using the MolDock SE algorithm, and the top five poses were retained. A grid with a radius of 15 Å and a resolution of 0.30 Å was generated centered on the crystallographic ligand positions in the selected proteins. The docked poses were subsequently analyzed using Discovery Studio Visualizer v21.1.0.20298.^18^

## Statistical analysis

After performing the normality test and verifying parametric sampling, the results obtained were analyzed by ANOVA, followed by Tukey’s post-hoc test, using Jamovi software, version 2.3.12. The values are expressed as mean ± standard deviation (SD). A significance level of 5% (p < 0.05) was considered.

## Results

### In vivo tests

The formalin test is widely used as an experimental model to evaluate pain mechanisms and the efficacy of antinociceptive agents, due to its ability to reproduce a biphasic response that reflects different nociceptive processes. It is a nonspecific test, related to the modulation of the response in receptors such as TRPV1, NMDA, and opioid receptors.^19^

**Figure 2:**
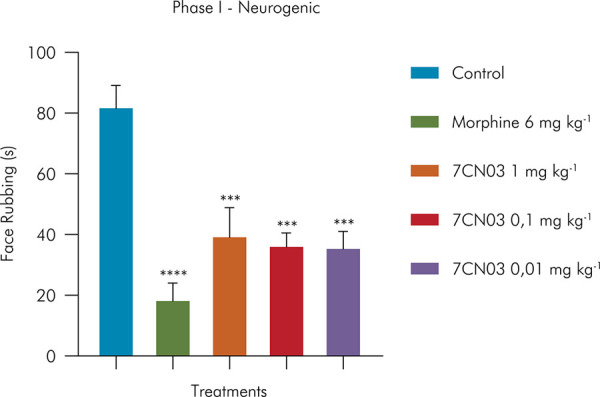
Effect of 7CN03 on formalin-induced orofacial nociception (neurogenic phase). Control (Tween 80 5% + saline, given orally), 7CN03 (1, 0.1 and 0.01 mg/kg, given orally), and morphine (20 mg/kg, given orally). Values were expressed as mean and SD (n= 6, per group). Statistical analysis: one-way ANOVA followed by Tukey’s test, *p < 0.05, **p < 0.01, ****p < 0.001: (7CN) = vs. control and (morphine) = vs. control.

In the formalin-induced nociception model, in the neurogenic phase, acute exposure to 7CN03 at doses of 1 mg/kg, 0.1 mg/kg, and 0.01 mg/kg significantly reduced facial rubbing behavior by 52% (39.83 ± 9.083), 55% (36.67 ± 3.870), and 56% (36.00 ± 5.027), respectively, compared to the control group. A significant reduction in facial rubbing by 77% (18.8 ± 5.2 s) was also observed for the group of mice treated with morphine (20 mg kg^-1^). These findings are shown in Figure 2.

**Figure 3:**
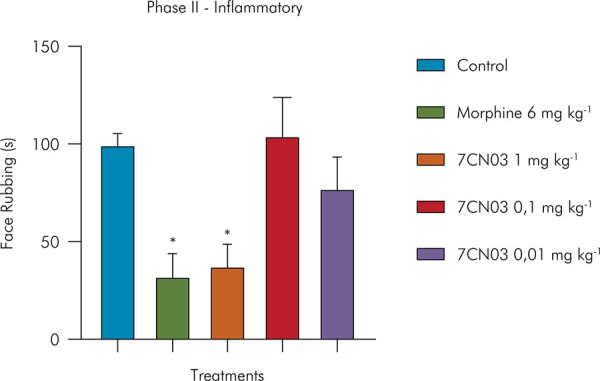
Effect of 7CN03 on formalin-induced orofacial nociception (inflamatory phase). Control (Tween 80 5% + saline, given orally), 7CN03 (1, 0.1 and 0.01 mg/kg, given orally), and morphine (20 mg/kg, given orally). Statistical analysis: one-way ANOVA followed by Tukey’s test, *p < 0.05, **p < 0.01, ****p < 0.001: (7CN) = vs. control and (MK-801) = vs. control.

In the inflammatory phase of the formalin test, only the 1 mg/kg dose of 7CN03 significantly reduced nociceptive behavior (37.50 ± 12.0 s) compared to the control group, equivalent to 62% less facial rubbing time. Morphine (20 mg kg^-1^) also promoted a significant reduction in orofacial nociception by 67% (32.3 ± 12.4 s), compared to the control group (99.6 ± 5.6 s). These findings are shown in Figure 3.

The induction of experimental nociception by glutamate allows us to evaluate the impact of new agents on the blockade of this excitatory pathway and on the modulation of associated receptors, especially NMDA, frequently implicated in synaptic plasticity and pain chronification.^20^ In this test, when compared to the control group, 7CN03 caused a significant reduction in orofacial nociceptive behavior (p < 0.05) at the three tested doses.

**Figure 4:**
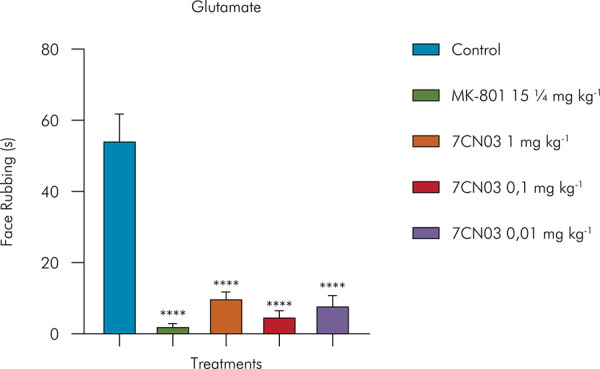
Effect of 7CN03 on glutamate-induced orofacial nociception. Control (Tween 80 5% + saline, given orally), 7CN03 (1, 0.1 and 0.01 mg/kg, given orally), and MK-801 (15 µg/kg, i.p). Values were expressed as mean and SD (n= 6, per group). Values were expressed as mean and SD (n= 6, per group). Statistical analysis: One-way ANOVA followed by Tukey’s test, *p< 0.05, **p < 0.01, ****p < 0.001: (7CN) = vs. Control and (morphine) = vs. Control.

As indicated in Figure 4, the group of mice treated with 7CN03 at doses of 1 mg/kg, 0.1 mg/kg, and 0.01 mg/kg significantly reduced their facial rubbing behavior, respectively, by 81% (10.17 ± 1.62), 90% (5.0 ± 1.50), and 85% (8.16 ± 2.57) compared to the control group (54.5 ± 7.0 s). The glutamate NMDA receptor antagonist MK-801 (15 µg kg^-1^) significantly reduced facial rubbing by 95% (2.3 ± 0.5 s).

The capsaicin-induced nociception model is particularly relevant for evaluating compounds that modulate the transient receptor potential vanilloid 1 (TRPV1), an ion channel highly sensitive to heat, protons, and chemical compounds such as capsaicin, thereby aiding the development of novel therapies for acute and chronic painful conditions associated with TRPV1 hyperactivity.^21^

**Figure 5:**
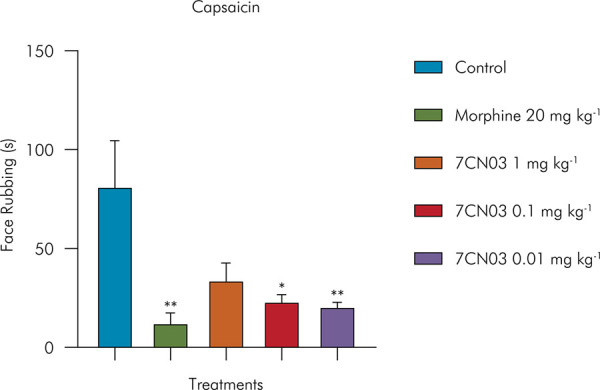
Effect of 7CN03 on capsaicin-induced orofacial nociception. Control (Tween 80 5% + saline, given orally), 7CN03 (1, 0.1 and 0.01 mg/kg, given orally), and morphine (20 mg/kg, given orally).

In the nociception induction tests with capsaicin, acute exposure to 7CN03 at doses of 0.1 mg/kg and 0.01 mg/kg significantly reduced facial rubbing behavior by 71% (23.40 ± 3.25) and 74% (20.80 ± 1.98), respectively, compared to the control group (81.6 ± 22 s). In this test, despite the reduction of nociceptive behavior by 58% (34.20 ± 8.47), the 1 mg/kg dose did not show a statistically significant difference when compared with the other doses. The group of mice treated with morphine (20 mg kg^-1^) significantly reduced facial rubbing by 84% (12.6 ± 4.8 s). These findings are presented in Figure 5.

### Molecular docking

The complex formed by 7CN03 and the µ-opioid, TRPV1, and NMDA receptors exhibited more negative scores compared to those formed with the controls, indicating a more favorable interaction with lower energy expenditure. These findings are presented in Table 2.

**Table 2:** MolDock scores (in Kcal/mol) and RMSD of the analyzed complexes.

Targets	Ligands	MolDock score	RMSD
µ-opioid receptor	7CN03	-97.00	0.14
	Morphine	-71.43	
δ-opioid receptor	7CN03	-112.31	0.28
	DPI-287	-141.16	
κ-opioid receptor	7CN03	-107.28	0.15
	MP1104	-138.26	
TRPA1	7CN03	-134.28	0.35
	AM-0902	-195.21	
TRPV1	7CN03	-87.79	0.66
	Capsazepine	-85.05	
NMDA receptor	7CN03	-104.86	-
	Esketamine	-80.87	

**Figure 6:**
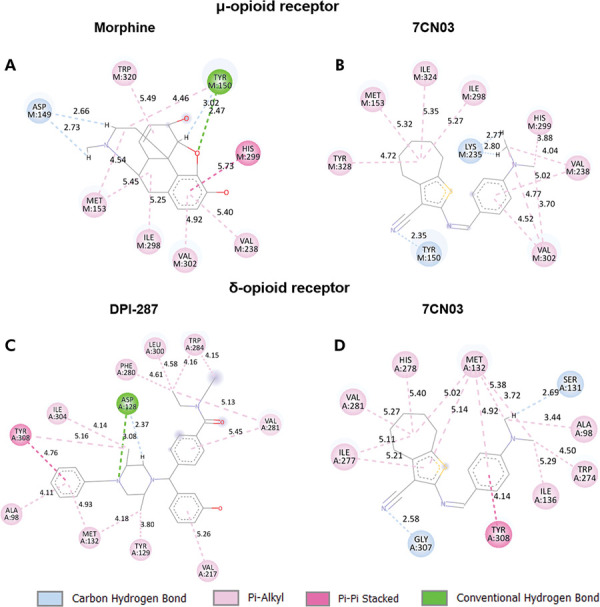
Interactions between 7CN03 and positive controls with µ and δ opioid receptors.

**Figure 7:**
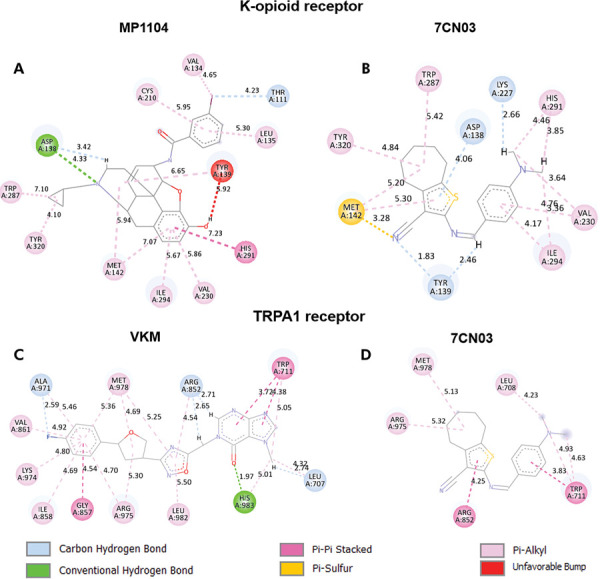
Interactions between 7CN03 and positive controls with κ-opioid and TRPA1 receptors.

**Figure 8:**
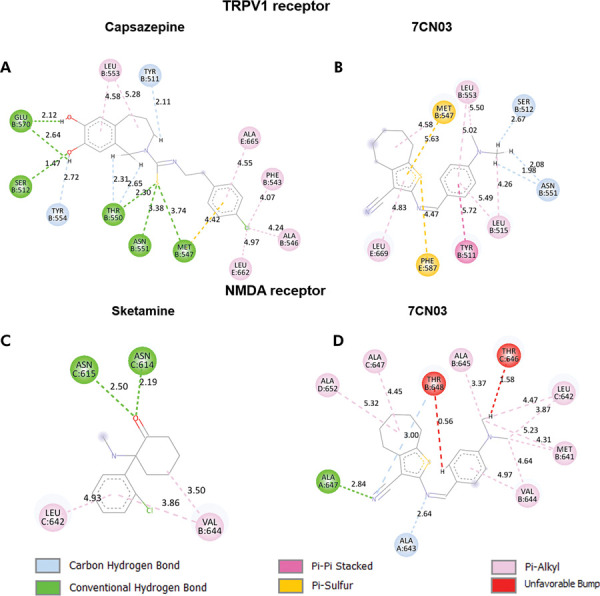
Interactions between 7CN03 and positive controls with TRPV1 and NMDA receptors.

Figures 6, 7, and 8 illustrate the main types of interactions between the tested compound and the amino acids of the evaluated receptors. The complex formed by 7CN03 and the µ-opioid receptor showed a more negative score (-97.00) compared to the complex formed with morphine (-71.43) (Table 2). The analysis of the involved interactions reveals common amino acids in both complexes: Met153, Ile298, Val302, and Tyr150 (Table 3 and Figs 6a,b).

**Table 3:** Types of interactions between ligands and residues of the analyzed targets, with residues shared by both ligands in boldface.

Ligand	π-alkyl	π-π stacking	Hydrogen bonding	Carbon-hydrogen bonding
µ-opioid receptor				
7CN03	Met153, Ile298, His299, Val302, Ile324, Tyr328.	-	-	Tyr150, Lys235
Morphine	Met153, Tyr150, Val238, Ile298, Val302, Trp320.	His299	Tyr150	Asp149, Tyr150
δ-opioid receptor				
7CN03	Ala98, Met132, Ile136, Trp274, Ile277, His278, Val281.	Tyr308	-	Gly307, Ser131
DPI-287	Ala98, Tyr129, Met132, Phe280, Val281, Trp284, Leu300, Ile304.	Tyr308	-	-

Regarding the δ-opioid receptor,^22^ the complex formed by the selective agonist DPI-287 exhibited a more negative score (-141.16) compared to that formed by the thiophene derivative 7CN03 (-112.31) (Table 4 and Figures 6c,d).

**Table 4:** Types of interactions between ligands and residues of the analyzed targets, with residues shared by both ligands in boldface.

Ligand	π-alkyl	π-π stacking	Hydrogen bonding	Carbon-hydrogen bonding	Unfavorable interactions	π-sulfur
κ-opioid receptor						
7CN03	Val230, Trp287, His291, Ile294, Tyr320	-	-	Lys227, Asp138, Tyr139	-	Met142
MP1104	Val134, Leu135, Tyr139 Met142, Cys210, Val230, Trp287, Ile294, Tyr320	His291	Asp138	Thr111	Tyr139	-
TRPA1 receptor						
7CN03	Leu708, Arg975, Met978	Trp711, Arg852	-	-	-	-
AM-0902	Leu707, Arg852, Ile858, Val861, Ala971, Met978, Lys974, Arg975, His983, Leu982	Trp711, Gly857	His983	Leu707, Arg852, Ala971	-	-

The N,N-diethylbenzamide portion of DPI-287 is capable of establishing hydrophobic interactions with the residues Val281, Phe280, Trp284, and Leu300 (Table 1), while the benzyl portion forms π-π stacking interactions with Tyr308. Finally, the methyl groups establish hydrophobic interactions with Met132, Tyr129, Ile304, and Tyr308 (Figure 6c).

7CN03, in turn, establishes the same type of interaction with Ala98, Met132, Val281, and Tyr308 (Table 3), but also contains two carbon-hydrogen bonds: one between the hydrogen of the N,N-dimethylaniline portion of 7CN03 and Ser131, and another between the nitrile group of the thiophene ring and Gly304 (Figure 6d).

As for the κ-opioid receptor complexes, the one formed with the agonist MP1104 presented a more negative score (-138.26) compared to that formed with 7CN03 (-107.28) (Table 2).

Hydrophobic interactions were observed between the cyclopropylmethyl group of MP1104 and the aromatic ring of the Tyr320 and Tyr287 side chains (Figure 7a).

The TRPA1 receptor^23^ formed a significantly more stable complex with the antagonist VKM (-195.21) compared to 7CN03 (-134.28) (Table 2). VKM also forms hydrogen bonds between the oxygen of the hypoxanthine portion and His983, as well as carbon-hydrogen bonds between the hydrogen of this same portion and Arg852. Additionally, the fluorine attached to the benzene ring also forms a carbon-hydrogen bond and a pi-alkyl interaction (Figure 7c).

On the other hand, 7CN03 only forms pi-alkyl interactions and pi-pi stacking, which, combined with a lower number of interactions compared to VKM, may explain the observed score values (Figure 7d). Despite establishing more interactions with the TRPV1 receptor,^24^ particularly through hydrogen bonds and π-alkyl interactions (Table 5), the antagonist capsazepine formed a complex with a score value similar to that of 7CN03: -85.05 for capsazepine and -87.79 for 7CN03 (Table 2).

**Table 5:** Types of interactions between ligands and residues of the analyzed targets, with residues shared by both ligands in boldface.

Ligand	π-alkyl	π-π stacking	Hydrogen bonding	Carbon-hydrogen bonding	Unfavorable interactions	π-sulfur
TRPV1						
7CN03	Leu515, **Leu553**, Leu669	**Tyr511**	-	**Ser512**, **Asn551**	-	**Met547**, Phe587
Capsazepine	Phe543, Ala546, Leu553, Leu662, Ala665	-	**Ser512**, Met547, Thr550, **Asn551**, Glu570	**Tyr511**, Tyr554, Thr550	-	**Met547**
NMDA						
7CN03	-	-	Ala647	Ala643, Thr648	Thr646, Thr648	-
Esketamine	Leu642, Val644	-	Asn614, Asn615	-	-	-

The thiophene ring of 7CN03 formed two π-sulfur interactions: one involving the sulfhydryl group of Met547 and the π electrons of the thiophene ring, and another between the thiophene sulfur and the benzene ring of Phe587 (Figure 8b).

Finally, the complex formed between NMDAR^25^ and 7CN03 exhibited a more negative score (-104.86) compared to that formed with esketamine (-80.87) (Table 2). These values may be explained by the fact that 7CN03 established more interactions with the receptor, such as a hydrogen bond between the nitrile group and Ala647, and a carbon-hydrogen bond between the imine linked to the thiophene ring and Ala643. However, there are unfavorable interactions between 7CN03 and the target: one between the hydrogen of the N,N-dimethylbenzylamine portion of 7CN03 and Thr646, and another one between the hydrogen of the benzene ring and Thr648 (Figures 8c,d), which may reduce the stability of the complex.

## Discussion

The objectives of this study were to evaluate the potential antinociceptive effects of the thiophene derivative 7CN03 using orofacial nociceptive models and to suggest binding targets for the observed effects. The results obtained from in vivo tests demonstrate orofacial antinociceptive activity, possibly related to the interaction between 7CN03and opioid, glutamatergic, and transient receptor potential vanilloid receptors, as indicated by in silico testing.

This investigation contributes to the development of a new analgesic drug, given the increasing morbidity and mortality particularly associated with currently used opioids.^6^

The antinociceptive effect of thiophene derivatives may be attributed to a combination of the properties of the thiophene ring and the substituents attached to it. The thiophene ring is an aromatic structure that confers chemical stability, lipophilicity, and the ability to interact with molecular targets, including proteins and receptors involved in nociception, such as TRPV1, NMDA, and opioid receptors. Furthermore, its planar conformation and sulfur-induced electronegativity make the ring a favorable core for π-π interactions and hydrogen bonds, which are essential for pharmacological activity.^26^ Not only this type of interactions, but also π-sulfur and carbon-hydrogen interactions between the thiophene ring and the coupling substituents were evidenced in the molecular docking findings.

The substituents on the thiophene ring are crucial for modulating antinociceptive activity. They can alter the lipophilicity and bioavailability of the compound, influencing its ability to cross biological membranes, such as the blood-brain barrier. They may also affect specific interactions with receptors, enzymes, and ion channels related to pain, and confer additional properties, such as antioxidant, anti-inflammatory, or free radical stabilization activities, which complement the antinociceptive effect.^27^

Previous studies have investigated thiophene derivatives using molecular docking to assess their pharmacological potential. Da Cruz et al.^7^ investigated the antinociceptive activity of the RMD86 derivative on COX-1 and COX-2, demonstrating that its binding sites exhibit similarities to those of meloxicam. Romeo et al.^28^ evaluated the analgesic profile of a series of thiophene derivatives such as TRPV1 receptor agonists, indicating that the thiophene core may contribute to compound selectivity by establishing hydrophobic interactions with the enzyme. Additionally, Dawood et al.^29^ analyzed a series of thiophene derivatives and observed that these compounds exhibit interactions with TNF-alpha similar to those of celecoxib, which may be related to the observed analgesic activity.

A previous study has reported that the yield of 7CN03 in the synthesis process exceeds 70%, indicating a positive aspect in the laboratory production system.^10^ In this study, 7CN03 was administered orally under rigorous methodological standards, including probabilistic sampling and blinding during the assays, representing an important strategy to meet the requirements of translational research, which considers the interplay between basic and clinical research, focusing on underlying scientific and operational principles.^30^

The susceptibility of the orofacial region to acute pain, resulting from exposure to algogenic substances that sensitize the studied receptors (capsaicin, formalin, and glutamate), provides an interesting strategy for pharmacological investigation aimed at discovering new therapeutic possibilities,^31^ especially considering that these algogenic agents are known for activating different types of nociceptors.

Orofacial pain in humans can result from inflammatory processes within the tissues, such as acute pulpitis and mucositis, as well as from chronic alterations involving temporomandibular joints and muscles. It is known that the mechanisms of nociceptive stimulus transmission to the trigeminal nerve occur through A-delta and C fibers present in the facial region.^32^ The neurogenic phase is characterized by the central nervous system processing of pain, triggered by the direct activation of C-type afferent nociceptors, the release of substance P, and other neuropeptides.^12^

One of the algogenic substances used in the in vivo tests was formalin, a substance capable of inducing the excitation of nociceptive neurons in the trigeminal nucleus, stimulating nociceptive fibers present in the orofacial region after its application. This is a valid and reliable biphasic evaluation method, consisting of a neurogenic phase followed by an inflammatory phase.^33^

The inflammatory phase, triggered by spinal cord stimulation after nociceptor and central neuron sensitization, involves the release of serotonin, histamine, prostaglandin E2 (PGE2), nitric oxide (NO), excitatory amino acids (glutamate and aspartate), and bradykinin.^34^ Previous investigations have demonstrated that thiophene derivatives, with chemical structures similar to those of 7CN03, were capable of reducing the quantity of inflammatory cytokines, such as IL-8, and exhibited antinociceptive and anti-inflammatory activities.^8^

The antinociceptive activity of 7CN03 may be associated with the reduction of inflammatory pain, but particularly with neurogenic pain, as better results were obtained in the neurogenic phase. Previous studies have reported that thiophene derivatives RMD86 (25, 50, and 100 mg/kg) and RBF(3)K (5 mg/kg) are capable of promoting antinociceptive effects in both phases of the formalin test in mice.^7,35^

Central-acting drugs act in both phases of the test, while peripheral-acting drugs (e.g.,, NSAIDs) inhibit only the inflammatory phase.^11^ Thus, the findings of the present study suggest that 7CN03 acts in both phases. The effect of 7CN03in the neurogenic phase, similar to that observed for morphine, may result from its interaction with opioid receptors, especially µ-opioid, given that the molecular docking study predicted a binding affinity of 7CN03 for this receptor.

According to Zhuang et al.,^36^ ohmefentanil, a potent μ-opioid receptor agonist, has the ability to establish additional interactions with residues Tyr150 and Asp149, which may explain its increased potency compared to fentanyl. The same amino acid residue forms a carbon-hydrogen bond between the N,N-dimethylaniline portion of 7CN03 and the Tyr150 residue, which may contribute to the activation of this receptor and a possible antinociceptive effect.

It is known that mutations in Tyr320 significantly reduce the potency of MP1104, a κ-opioid receptor ligand, for G protein signaling (mainly Gi), while mutation in Tyr287 selectively reduces the potency of β-arrestin 2 recruitment by MP1104, a protein frequently associated with adverse effects such as dysphoria, sedation, and lower reward threshold, which may lead to depression-like symptoms.^37^ In this study, we observed that Tyr320 and Tyr287 residues established the same types of interactions (π-alkyl) in the complex formed with 7CN03, suggesting that this thiophene derivative may have similar activity to MP1104 regarding G protein cellular signaling.

The residue M142, in turn, is located in an aromatic cluster involved in the activation of various G protein-coupled receptors.^38^ While MP1104 establishes π-alkyl interactions, 7CN03 forms stronger π-sulfur bonds between the nitrile group of the thiophene derivative and the sulfur of the amino acid side chain. These data further support that 7CN03 may act in cellular signaling similarly to MP1104.

Glutamate is an amino acid with an important role in excitatory neurotransmission, and its effects are mediated by ionotropic (NMDA and AMPA) or metabotropic (mGluR) receptors. The activation of these receptors promotes modulation of cyclic adenosine monophosphate (cAMP), which triggers the propagation of nerve impulses through sodium ion influx. Rapid synaptic transmission occurs from the activation of ionotropic receptors, as they are associated with the opening of channels for Ca2+ ions.^39^ Our findings from the molecular docking study provided evidence of strong interaction of 7CN03 with the NMDA receptor. The involvement of the N,N-dimethylaniline portion and the thiophene core of 7CN03 in the establishment of several relevant intermolecular interactions highlights the importance of these two groups (N,N-dimethylaniline portion and the thiophene core) for potential biological activity.

This possible interaction of 7CN03 with the NMDA receptor helps to explain the observed antinociceptive effect, as glutamate can excite nociceptive afferents supplying the facial musculoskeletal tissues, eliciting a short-lived nociceptive response.^40^ Consistently, in humans, pain and mechanical hyperalgesia induced by glutamate injection into the masseter muscle are attenuated by treatment of this muscle with ketamine, which blocks NMDA receptors, indicating that peripheral NMDA receptors contribute to inflammation, nociception, and hyperalgesia in the masseter muscles.^40^

Capsaicin (8-methyl-N-vanillyl-trans-6-zonisamide) is a chemical substance extracted from red pepper of the Capsicum genus, capable of exciting nerve endings through the activation of transient receptors from the vanilloid family, especially TRPV1, which is responsible for generating a sensation of heat in neurons, resulting in pain.^21^

The capsaicin-induced nociception model is useful for studying the mechanisms triggered by trigeminal nociception, as it mimics acute pain following a harmful stimulus. Capsaicin, when applied to the skin, muscles, and other tissues, has been shown to produce inflammation and sensitizes trigeminal and spinal nociceptive afferents, as well as dorsal horn neurons.^12^ Vanilloid receptor antagonists have been studied in murine models of oral ulcerative mucositis, a condition that causes intense pain during eating and speaking, resulting in a low quality of life for cancer patients undergoing chemoradiotherapy.^41^

It is known that, regardless of how the drug effect occurs, whether through binding or chemical interaction, drug concentration at the site of action controls the effect. Nevertheless, the response to concentration can be complex and is often nonlinear, as observed in the capsaicin test, which indicated that the best antinociceptive effect of 7CN03 occurred at the intermediate dose. This effect may be explained by factors related to pharmacokinetic aspects.^42^

The antinociceptive effect of 7CN03 may also be attributed to its interaction with the TRPV1 receptor, which, when activated by capsaicin, promotes cation influx and produces depolarization of the cell membrane. The transmission of the impulse resulting from this activation may occur through interaction with various mediators, such as tachykinins, substance P, excitatory amino acids, nitric oxide, and other pro-inflammatory mediators.^43^ Thiophene derivatives may exert their antinociceptive effects through a mechanism of modulation of the TRPV1 receptor.^44^

TRPV1 and TRPA1 are expressed in approximately 25% and 10% of masseter afferents, respectively.^41^ Furthermore, previous studies have suggested that the activation of the TRPV1 receptor in the facial skin of rats influences nociceptive responses to harmful thermal and mechanical skin stimuli, inducing neuroplastic changes in the caudal subnucleus of the trigeminal brainstem.^45^ The in silico analysis indicated a predicted affinity of 7CN03 for TRPV1, with a binding value close to that observed for the antagonist.

Although the findings of this study are promising, certain limitations should be taken into account. First, the experiments were conducted using animal models which, despite being widely employed for investigating nociceptive mechanisms, may not fully capture the complexity of orofacial pain in humans. Furthermore, molecular docking analysis suggested possible interactions with opioid, TRPV1, and NMDA receptors; however, these findings need to be confirmed through experiments that directly assess the binding of 7CN03 to these biological targets and its functional effects. Another limitation is the lack of a detailed pharmacokinetic analysis, as factors such as bioavailability, metabolism, and potential drug interactions could influence the efficacy and safety of 7CN03. Addressing these limitations in future research will allow for a more precise evaluation of the clinical applicability of 7CN03.

## Conclusion

7CN03 appears to hold significant promise for the development of novel formulations for the treatment of orofacial pain. Our findings suggest that 7CN03 has an orofacial antinociceptive effect, with a probable target of action involving opioid, glutamatergic, and vanilloid receptors, and that these mechanisms need to be investigated in future in vivo trials. Elucidation of the mechanism of action and pharmacokinetics through future research is extremely important for understanding the pharmacological effect and subsequent evaluation of the efficacy of 7CN03 in clinical trials to determine its clinical application.

## Data Availability

After publication the data will be available on demand to the authors.
